# A phase II multicenter randomized study to evaluate the safety and efficacy of combining thermotherapy and a short course of miltefosine for the treatment of uncomplicated cutaneous leishmaniasis in the New World

**DOI:** 10.1371/journal.pntd.0010238

**Published:** 2022-03-07

**Authors:** Liliana López, Braulio Valencia, Fiorela Alvarez, Ana Pilar Ramos, Alejandro Llanos-Cuentas, Juan Echevarria, Iván Vélez, Marina Boni, Joelle Rode, Juliana Quintero, Alejandra Jiménez, Yulied Tabares, Claudia Méndez, Byron Arana

**Affiliations:** 1 PECET—Programa de Estudio y Control de Enfermedades Tropicales, Facultad de Medicina, Universidad de Antioquia, Medellín, Colombia; 2 Unidad de Leishmaniasis y Malaria, Instituto de Medicina Tropical Alexander von Humboldt, Universidad Peruana Cayetano Heredia, Lima, Peru; 3 Drugs for Neglected Diseases *initiative* (DND*i*), Rio de Janeiro, Brazil; 4 Dirección de Sanidad, DISAN, Colombian Army, Bogotá, Colombia; 5 Drugs for Neglected Diseases *initiative* (DND*i*), Geneva, Switzerland; 6 Kirby Institute, The University of New South Wales (UNSW Sydney), Sydney, Australia; RTI International, UNITED STATES

## Abstract

**Background:**

Systemic pentavalent antimonials, mainly meglumine antimoniate, continue to be the first-choice drugs for treatment of cutaneous leishmaniasis (CL) despite their toxicity, difficulty of administration and high cost. In the search for therapeutic alternatives, combining two treatment interventions has emerged as a potential alternative to either reduce the use of antimonials with the associated toxicities, or to increase efficacy. Here, we report the results of a recently completed trial assessing the efficacy and safety of a combination of thermotherapy (TT) plus a short course of miltefosine (MLT) for the treatment of uncomplicated CL in Colombia and Peru.

**Methods:**

A multicenter, randomized, evaluator-blinded, phase II, controled clinical trial was conducted. Adult volunteers with a parasitologically confirmed diagnosis of uncomplicated CL were randomly allocated to receive either a single session of TT or a combination of TT plus a short course of MLT (3 weeks). Therapeutic response outcomes and safety were assessed.

**Results:**

130 subjects were included in the study, of whom 64 were randomly assigned to the TT arm and 66 to the TT + MLT arm. Cure at 3 months’ follow-up was achieved in 57.8% (n = 37) and 80.3% (n = 53) in the TT and TT + MLT groups, respectively, in the intention to treat analysis. The TT + MLT regimen was better that TT alone (p = 0.0055). The presence of vesicles at the site of heat application was the most common adverse event reported associated with the use of TT; while vomiting (31.8%) and elevation of liver enzymes (28.8%) were the most frequent adverse events reported associated with the use of MLT.

**Conclusion:**

The combination of TT plus a short course of MLT was shown to be significantly better than TT alone for the treatment of uncomplicated CL in the New World.

**Trial registration:**

Registered in clinicaltrials.gov NCT02687971.

## Author summary

Cutaneous leishmaniasis is a disease caused by *Leishmania* parasites transmitted by sandflies. It is a complex and neglected disease. Generally, it manifests as ulcers, nodules, or other lesions, mainly on the face and extremities. An estimated 0.7–1 million new cases occur annually in around 88 countries in South and Central America, Africa, Europe, the Middle East, and Asia, with the appearance of new outbreaks in different regions of the world.

For more than 70 years, pentavalent antimonials have been the first line treatment, despite their toxicity, varying efficacy and need of administration of painful injections for 20 days or more.

There is limited availability of effective and safe treatment alternatives and the strength of scientific evidence on the efficacy and safety of the different schemes is variable, limiting the control of the disease from a therapeutic approach. Hence the need for effective, safe, low-cost treatment alternatives with non-invasive routes of administration.

This article reports the results of a clinical trial aiming to evaluate a combined therapy with two therapeutic alternatives (miltefosine and thermotherapy) approved for the treatment of cutaneous leishmaniasis, comparing it with thermotherapy alone. In this study, the combined therapy was significantly better than the monotherapy in managing cutaneous leishmaniasis.

## Introduction

Of the different clinical forms of leishmaniasis, cutaneous leishmaniasis (CL) is the most frequent. An estimated of 0.7–1 million new cases from around 88 countries are estimated to occur annually [1,2]. CL is caused by over 15 different species of the protozoan parasite *Leishmania*, with *L*. *tropica* in the Old World and *L*. *braziliensis* in the New World being the most important species, given the severity and duration of the disease and the difficulty of treating them [3,4]. Typically CL lesions begin as a papule at the site of a sandfly bite that enlarges to a nodule, and ulcerates over a period of 1–3 months [5].

Between 2001 and 2019, a total of 1,028,054 new cases of CL or mucocutaneous leishmaniasis (MCL) in the Americas were reported to the Pan American Health Organization (PAHO). In 2019, of the 41,617 new cases of CL and MCL, 75% were reported by Brazil (15,482), Colombia (5,097), Peru (5,349), Nicaragua (3,321) and Bolivia (2,050)[6]. In Colombia, most cases are associated with infections caused by *L*. *panamensis* and, to a lesser extent, by *L*. *braziliensis*. Meanwhile, in Peru, the predominant species are *L*. *braziliensis* and *L*. *peruviana* [1,5].

Pentavalent antimonials, mainly meglumine antimoniate at doses of 20 mg Sb^v^/kg/day for 20 days, continue to be the first-choice drugs to treat CL despite their toxicity, difficulty of administration and high cost. Alternative treatment regimens, including miltefosine, pentamidine, isethionate, amphotericin B and topical therapies, such as intralesional antimonials, heat therapy or cryotherapy, are less commonly used [7,8].

Combining two treatment interventions has emerged as a potential alternative to either reduce the use of antimonials and their associated toxicities or to increase efficacy. The most used combination so far is the association of intralesional antimonials with liquid nitrogen, which in the Old World has shown to be more effective than using either intervention alone [9–11]. In the New World, different combinations have been evaluated, mostly associating systemic meglumine antimoniate with other systemic or local treatments [12–15] with variable results ranging from no difference between mono and combination therapy to better efficacy with the combination.

Thermotherapy, especially generated via radio frequency waves (ThermoMed) has been widely tested for CL in both Old and New Worlds. The procedure and the device were approved for commercialization by the United States Food and Drug Administration (FDA) for the treatment of CL in 2007. Thermotherapy was also included in the list of recommended treatments for uncomplicated CL cases by WHO in 2010 [5] and by PAHO in 2013 [16]. Heat application (50°C for 30 seconds) usually requires the use of local anaesthesia [17–19]. In 2014, the FDA approved the use of oral miltefosine at a dose of 2.5mg/kg/day for 28 days for the treatment of CL in the New World for lesions due to *L*. *braziliensis*, *L*. *panamensis* and *L*. *guyanensis* only. In studies conducted in the Americas, the effectiveness of oral miltefosine varies from 90% when evaluated in infections caused by *L*. *panamensis* to <60% for CL caused by *L*. *braziliensis* and *L*. *mexicana* [5,20]. Although its oral administration facilitates field implementation, it is limited by the 28-day treatment duration, gastrointestinal adverse events (AE) and teratogenicity, which limits its use in women who are either pregnant or of childbearing potential, unless contraception is provided for at least 5 months after the end of treatment. [4].

Here, we report the results of a recently completed phase II, randomized multicenter trial assessing the efficacy and safety of a combination of thermotherapy (TT) plus a short course of miltefosine (MLT) for the treatment of uncomplicated CL in Colombia and Peru.

## Methods

### Ethics statement

In Colombia, the protocol was approved by the Research Ethics Committee of the IPS Universitaria, by the Ethics Committee of the Military Hospital of the Colombian Army and by the National Regulatory Authority (Instituto de Vigilancia de Medicamentos y Alimentos—INVIMA); in Peru the protocol was approved by the Universidad Peruana Cayetano Heredia Ethics Committee and by the Peruvian National Institute of Health. The study was carried out according to international norms of good clinical practice. Before entry into the study, investigators obtained written informed consent from all participants. The trial was conducted following Good Clinical Practice standards.

#### Study design and sites

This is a randomized, multicenter, evaluator-blinded, phase II, clinical trial, registered at clinicaltrials.gov NCT02687971 (https://clinicaltrials.gov/ct2/show/NCT02687971).

Study subjects attended the PECET Clinic in Medellín, Colombia and the Leishmaniasis and Malaria Unit of Cayetano Heredia National Hospital in Lima, Peru. In Colombia, both civilian and military patients were included.

### Population

*Inclusion / exclusion criteria*: subjects who met the following criteria were included in the study: male or female, aged ≥18 and ≤60 years old, confirmed parasitological diagnosis of CL, subjects with ≤ 4 CL lesions of ≥ 0.5 cm and ≤ 4 cm (longest diameter) not located on the ear, face, close to mucosal membranes, joints, or on a location that in the opinion of the principal investigator would make the application of TT difficult. Subjects were excluded for the following reasons: females with a positive serum pregnancy test, breast-feeding or of childbearing potential who do not agree to take appropriate contraception during the treatment period and until day 90 after the onset of treatment; clinically significant medical problems as determined by history or laboratory studies; previous use of antileishmanial drugs (within 8 weeks); or abnormal laboratory values at baseline (serum creatinine above normal level; alanine aminotransferase (ALT) / aspartate aminotransferase (AST) 3 times above normal range).

Proof of infection was documented either through microscopic identification of amastigotes in stained lesion tissue, the demonstration of motile promastigotes in aspirate cultures, or demonstration of *Leishmania* by polymerase chain reaction (PCR) following previously published protocols [21,22]. In Peru, a filter paper lesion impression method was used for *Leishmania* species identification, as previously described [23].

### Interventions

Subjects were randomly allocated to receive TT (one session, 50°C for 30") or TT (one session, 50°C for 30") plus MLT 2.5 mg/kg/day for 21 days.

The 21-day course for MLT was chosen based on the results of a Phase II trial conducted in Colombia showing that a mean dose of miltefosine of 133 mg/day for 20 days resulted in a cure rate of 82% [24] and of a report showing that daily administration of 100 mg/day (2.5 mg/kg of body weight/day for 28 days) resulted in a mean maximum concentration of drug in serum of 70,000 ng/ml at day 23 of treatment [25].

Thermotherapy administration: local heat was generated using a device manufactured by Thermo-Med Technologies, Inc. (Phoenix, Arizona, USA). The lesions were cleansed, moistened for at least 10 minutes with normal saline and then anesthetized with 2% intralesional lidocaine before administration of thermotherapy. Heat was applied locally with a handset that includes two electrodes which were placed on the lesion(s) until the entire lesion was covered, including the affected areas around the ulcer. Patients were instructed to use clorixin gel and to dress their lesion after receiving thermotherapy and for up to three days to decrease the effects of burning and the risk of secondary infections.

Miltefosine was administered as follows: subjects weighing ≥30 to ≤45kg, received 50 mg twice a day (100 mg /day) whilst subjects above 45kg, received a 50 mg capsule three times per day (150 mg/day). Subjects were instructed to take the miltefosine capsules after meals to avoid gastric problems associated with the drug.

Participants assigned to the TT+MLT arm had their liver enzyme and creatinine levels evaluated at baseline and end of treatment (D21), the latter only for participants assigned to the combined treatment arm.

*Rescue therapy*: meglumine antimoniate at doses of 20 mg Sb^V^ /kg body weight per day for 20 days was provided free of charge to all subjects who were declared as treatment failures and to those who, for whatever reason, decided to withdraw from the study.

### Follow-up and outcomes

All subjects had a follow-up visit 24 hours after TT treatment and thereafter at day 7, 14, 21, 45±5 days, 63±5 days 90±15 days and on day 180±15 days. The following treatment outcomes were applied:

- Initial cure: complete re-epithelialization of all ulcerated lesions or flattening and/or no signs of induration of the non-ulcerated lesion(s) on day 90.
- Final cure: initial cure and no relapse by day 180.
- Relapse: lesion that achieved 100% re-epithelialization/flattening on day 90 that subsequently reopened/indurate by day 180.
- Failure for ulcerated lesions: <50% re-epithelialization of lesion as compared to day7 by nominal day 63 or <100% re-epithelialization of the lesion(s) area(s) on nominal day 90; or an increase of ≥ 100% of the ulcer area compared to the area at day 7 at any time before day 90.
- Failure for non-ulcerated lesions: <50% improvement/reduction of induration/flattening of the lesion as compared to day 7 by nominal day 63; persistence of induration of the lesion(s) on nominal day 90; or an increase of ≥ 100% of the induration area compared to the area at day 7 at any time before day 90. Finally, relapse of the lesion any time between day 90 and day10.

The primary endpoint was defined as *t*he proportion of initial clinical cure for each regimen measured at Day 90. Secondary endpoints were the proportion of final cure, the frequency, severity and seriousness of AEs, time to lesion healing and cure rates by *Leishmania* species, by treatment group.

Patients were actively followed up and no incentives to come back for follow-up visits were provided.

Photos of all lesions were taken at different times. Clinical experts on CL evaluated the lesions. First, a blinded assessment of the lesion was performed on site at day 90 and day 180 visits by a clinician. Second, assessment of the lesion photographs at day 1, day 90 and day 180 visits was performed by an independent DSMB expert. If there was a discrepancy between the assessments performed by the site clinician and the Data Safety Monitoring Board (DSMB) expert, a third expert was asked to review the photos and a final decision was taken based on the majority of votes (two out of three) and used to classify the subject´s outcome.

Considering that TT application can increase the lesion size, the percentage of re-epithelialization/flattening of the lesion(s) was calculated by comparing the size of the ulcer/induration at D7 against the size at the follow-up visit. After cleaning the lesion and removing the crust (if applicable), measurements were taken in two perpendicular directions using an electronic caliper. The area of ulceration was calculated using the area calculation for an ellipse as follows: Area = A/2*B/2*π mm^2^, where A = longest diameter of ulceration in mm; B = perpendicular to “A” diameter of ulceration in mm and π = 3.14.

### Sample size

Based on reports, the efficacy of thermotherapy alone in the New World is around 65–70% and, in the absence of information about the efficacy of the proposed combination, a sample size of 118 subjects (59 TT and 59 TT + MLT) was estimated to have an 80% chance of detecting, at a significance level of 5%, an increase in the primary outcome measure from 70% in the TT alone group to 90% in the combination group. Taking into account an estimated 10% loss during follow-up, six more subjects were added, resulting in a sample size of 65 subjects per regimen, or 130 subjects in total.

### Randomization process

A computer-generated randomization code using a 1:1 allocation ratio was used to assign subjects to either TT or TT + MLT. Randomization was centralized and only the study coordinators had access to the password to generate the treatment allocation.

### Statistical analysis

The participant’s baseline characteristics were analyzed by treatment group and the results presented as summary statistics. Intention-to-treat (ITT) and per-protocol (PP) analyses were used to determine the efficacy of treatments. Categorical variables were presented as counts and percentages. Given the distribution of the variables, the Mann-Whitney U test was used for analysis of continuous data. Patients with missing data that made it impossible to determine whether they were clinical cures were considered failures in the ITT analysis. All data were presented separately by treatment group, and by local and systemic AEs. AEs were coded using the most recent version of the Medical Dictionary of Regulatory Activities (MedDRA) preferred terms and grouped by system organ class (SOC) designation. All data were analyzed by treatment group, and by local and systemic AEs.

## Results

### Patient characteristics

**Fig 1:**
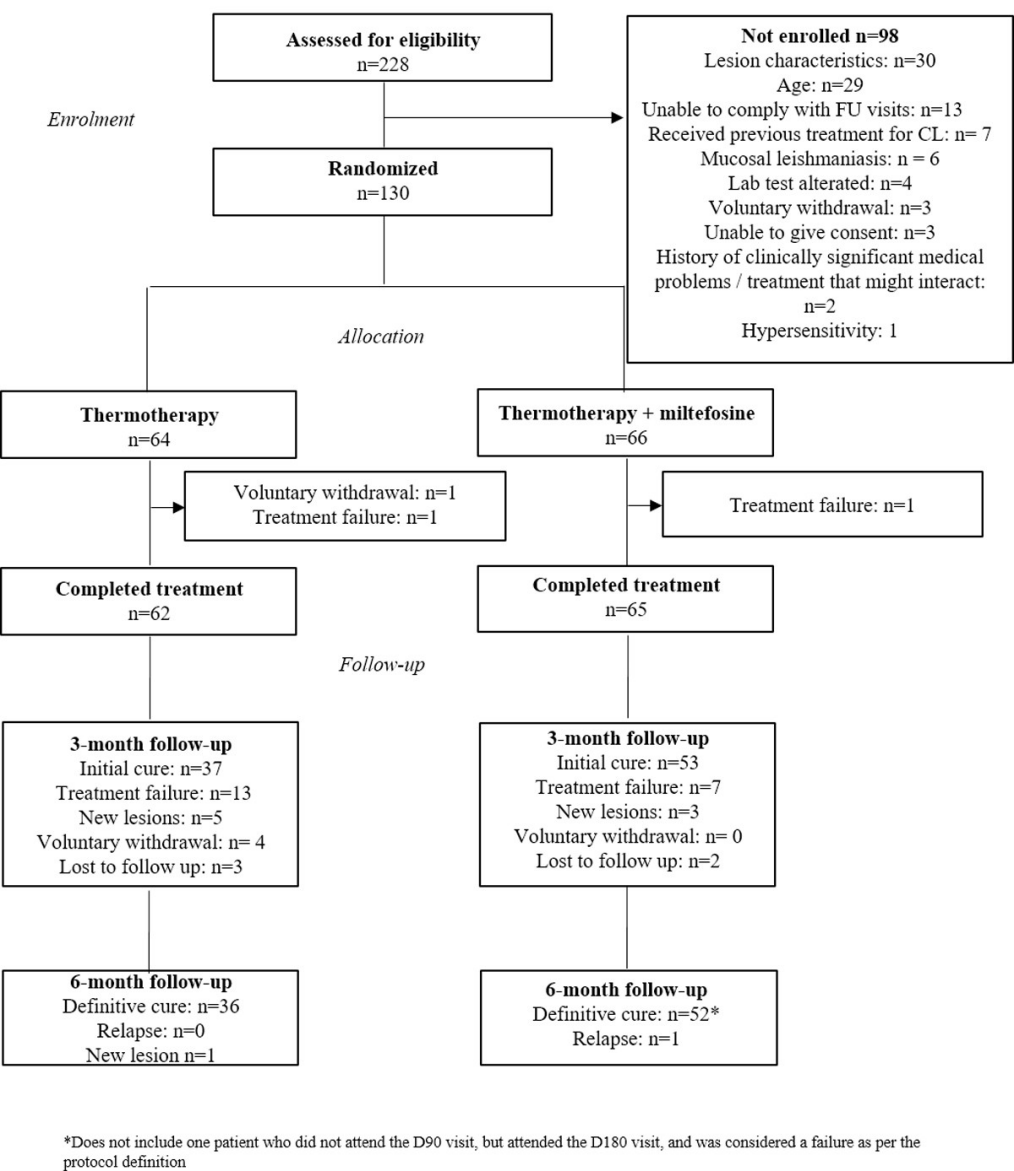
Flow diagram of study participants.

The study was carried out between December 2016 and February 2019. 130 subjects (65 volunteers per research center) were randomly assigned to one of the two treatment arms TT (n = 64) and TT + MLT (n = 66). In total, 91 subjects (37 TT and 54 TT + MLT) completed the study follow-up. One relapse occurred in the TT + MLT group. The description of treatment failures, voluntary withdrawals, and loss to follow-up are shown in Fig 1.

Mix raced males under 43 years of age accounted for at least 75% of the study population. *L*. *panamensis* was the main species responsible for infection in 30% of the cases. In both treatment groups, the majority of participants presented a single lesion (70.8% in total), and the majority of lesions were ulcerated: 88.2% and 96.9% for the TT and TT + MLT groups, respectively (Table 1).

**Table 1:** Baseline characteristics of volunteers.

Characteristic	TT (N = 64)	TT + Miltefosine (N = 66)	Total (N = 130)
**Gender n (%)**			
Female	16 (25)	14 (21.2)	30 (23.1)
Male	48 (75)	52 (78.8)	100 (76.9)
**Age** (Years) Median (IQR)	32.3 (25, 41.6)	34.3 (24.5, 42.8)	32.5 (24.8, 42.1)
**Race/Ethnicity n (%)**			
Black	0 (0.0)	3 (4.5)	3 (2.3)
Mixed	57 (89.1)	58 (87.9)	115 (88.5)
Other	1 (1.6)	2 (3.0)	3 (2.3)
White	6 (9.4)	3 (4.5)	9 (6.9)
***Leishmania* species n (%)**			
* L*. *braziliensis* (*Lb*)	7 (10.9)	13 (19.7)	20 (15.4)
* L*. *panamensis* (*Lp*)	20 (31.2)	20 (30.3)	40 (30.8)
* L*. *peruviana (Lpe)*	5 (7.8)	3 (4.5)	8 (6.1)
* L*. *braziliensis/L*. *peruviana (*mixed infection)	9 (14.1)	11 (16.7)	20 (15.4)
* *Other	7 (10.9)	3 (4.5)	10 (7.7)
No species identification	16 (25.0)	16 (24.2)	32 (24.6)
**Ulcer information**			
* * **Number ulcerated lesions (%)**	75 (88.2)	94 (96.9)	169 (92.9)
* ***Size** (mm^2^) Median (IQR)	95.1 (39.1, 186.4)	105.2 (50.9, 257.7)	102.1 (40.8, 227.4)
* ***Numbe**r	10 (11.8)	3 (3.1)	13 (7.1)
* ***Size** (mm^2^) Median (IQR)	61.6 (35.9, 384.0)	102.9 (12.0, 220.8)	71.4 (35.9, 284.7)
**Anatomical location n (%)**			
Head and neck	3 (3.5)	4 (4.1)	7 (3.8)
Thorax	4 (4.7)	10 (10.3)	14 (7.7)
Upper limbs	53 (62.3)	48 (49.5)	101 (55.5)
Lower limbs	25 (29.4)	35 (25.8)	60 (33)
**Number of lesions by patient n (%)**			
One	47 (73.4)	45 (68.2)	92 (70.8)
Two	14 (21.9)	14 (21.2)	28 (21.6)
Three	2 (3.1)	4 (6.1)	6 (4.6)
Four	1 (1.6)	3 (4.6)	4 (3.1)

Me (IQR): Median and interquartile range.

### Efficacy

Table 2 shows the results of the efficacy analyses. In the ITT analysis, the initial cure rates were 57.8% (n = 37) and 80.3% (n = 53) in the TT alone and the TT + MLT groups, respectively. Final cure rates were achieved by 56.3% (n = 36) and 78.8% (n = 52) in the TT and TT + MILT groups, respectively (p ≤ 0.05).

**Table 2:** Efficacy outcomes.

Analysis type		TT	TT + MLT	p value
**Initial cure (D90)**	**ITT**	37/64 (57.8%)	53/66 (80.3%)	p = 0.0055
	**PP**	37/56 (66.1%)	53/62 (85.5%)	p = 0.013
**Final cure (D180)**	**ITT**	36/64 (56.3%)	52/66 (78.8%)	p = 0.006
	**PP**	36/56 (64.3%)	52/62 (83.9%)	p = 0.014

TT: Thermotherapy. TT + MLT: Thermotherapy + Miltefosine

Five subjects, 3 (4.7%) and 2 (3.0%) in the TT and TT+MLT groups, respectively, were lost during the follow up period, whilst 9 (6.9%) developed a new lesion (TT n = 6 and TT + MLT n = 3) and were, therefore, removed from the study, declared as failures and administered rescue treatment (Fig 1).

**Fig 2:**
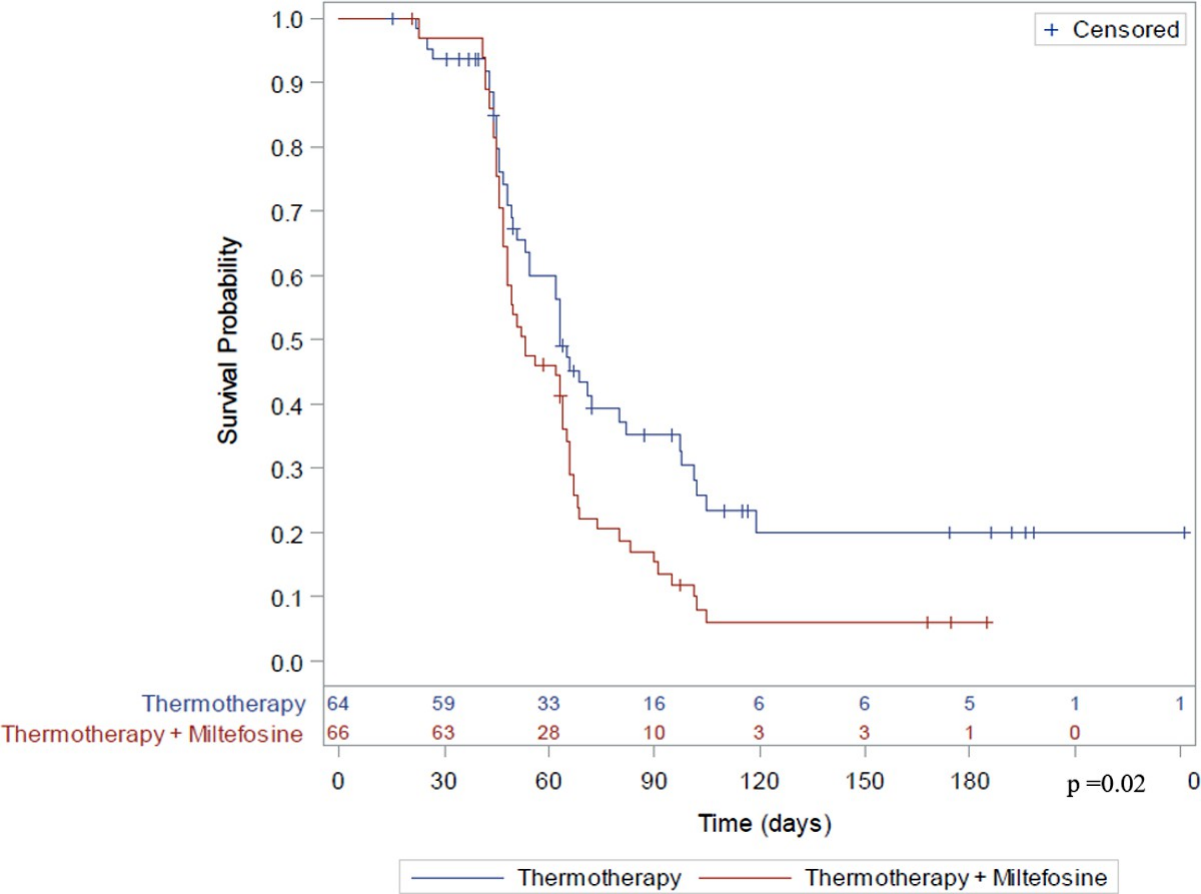
Survival analysis of time to healing of lesions.

The mean time for the lesions to heal was significantly lower (p = 0.02) in the TT + MLT group (61.2 days; 95% CI 55.9–66.5 days) than in the TT alone group (74.1 days; 95% CI 65.8–82.4 days) (Fig 2).

Although the numbers were low and the study was not powered to find differences in cure rates between different species of *Leishmania*, we observed that subjects with lesions due to *L*. *braziliensis* and/or *L*. *peruviana* responded better to TT + MLT (20/27 = 74.1%) than to TT alone (9/21 = 42.8%), although treatment response was lower than in subjects with lesions due to *L*. *panamensis* in both treatment groups (TT + MLT 16/20 = 80%; TT alone 12/20 = 60%) (Table 3).

**Table 3:** Final cure rate by *Leishmania* species and treatment group.

*Leishmania* Species n (%)	TT	TT + MILT	Total
*L*. *peruviana*	1/5 (20.0)	2/3 (66.7)	3/8 (37.5)
*L*. *braziliensis*	2/7 (28.6)	7/13 (53.8)	9/20 (45.0)
*L*. *braziliensis/ L*. *peruviana*	6/9 (66.7)	11/11 (100.0)	17/20 (85.0)
*L*. *panamensis*	12/20 (60.0)	16/20 (80.0)	28/40 (70.0)
Other	4/7 (57.1)	2/3 (66.7)	6/10 (60.0)
*Unidentified Leishmania* sp.	11/16 (68.8)	14/16 (87.5)	25/34 (73.5)

### Safety

Ninety two percent of subjects in the TT group and 100% in the TT+MLT group reported one or more AE (Table 4). Presence of vesicles at the site of heat application was the most commonly reported AE associated with the use of TT (Table 4). Most TT-related AE were reported during the first 24 hours after the application of local heat. Vomiting (31.8% of subjects) and elevation of liver enzymes -AST/ALT- (28.8% of subjects) were the most frequently reported AEs associated with the use of MLT (Table 4). With the exception of edema, there was no difference in local adverse reactions between the two treatment groups. All subjects reporting AEs recovered without any complications.

**Table 4:** Incidence of local adverse events and laboratory analysis by treatment group.

	TT (N = 64)	TT + MILT (N = 66)	p value
**Number of AE reported**	142 (59 subjects = 92.2%)	234 (66 Subjects = 100%)	0.000
**Related to thermotherapy**	79	74	0.000
**Related to miltefosine**	NA	114	-
**Local adverse reactions (%)**	**# of subjects (%)**	**# of subjects (%)**	
**Pain**	9 (14.1)	3 (4.5)	0.06
**Erythema**	18 (28.1)	19 (28.8)	0.93
**Local oedema**	20 (31.3)	18 (27.3)	0.62
**Oedema outside the lesion**	11 (17.2)	3 (4.5)	0.02
**Vesicles**	52 (81.3)	55 (83.3)	0.76
**Local infection**	4 (6.2)	3 (4.5)	0.67
**Gastrointestinal disorders (%)**	**# of subjects (%)**	**# of subjects (%)**	
**Vomiting**	0	21 (31.8)	-
**Nausea**	0	16 (24.2)	-
**Abdominal pain**	0	9 (13.6)	-
**Diarrhoea**	0	3 (4.5)	-
**AST / ALT elevation**	NA	19 (28.8)	-
**Creatinine elevation**	NA	2 (3.0)	-

NA = not applicable. AST: aspartate aminotransferase. ALT: alanine aminotransferase

Of the subjects included in the TT + MLT group, 56.1% (n = 37) reported having missed at least one dose of MLT, including one (1.5%) for AST/ALT elevation and n = 10 (15.2%) due mostly to gastrointestinal AEs associated with the medication.

During the study period, 3 serious AE were reported: an episode of urolitiasis in a subject in the TT group, and hypoglycemia and a skin lesion due to a road traffic accident in two subjects in the TT + MLT group; none of these were considered related to the study treatments.

## Discussion

In this first trial of a combination of one application of local heat plus 3 weeks of oral miltefosine, all study participants received their treatment according to which group they were assigned at the time of randomization. The study demonstrated that a single application of local thermotherapy in combination with 3 weeks of oral miltefosine is safe and delivers a higher cure rate than TT alone. Similarly, the time to heal for lesions treated with the combination was shorter than for lesions treated with TT alone.

Various combinations of systemic and topical treatments have been tested for CL with the aim of increasing the efficacy of local therapies and reducing the AE associated with systemic treatment alone, by using a reduced dose. Until now, the most frequently used combination was intralesional injection of antimonials alternating with local applications of liquid nitrogen. This combination has been shown in the Old World to be more effective that either treatment alone [26]. Other combinations have also been tested, but include therapeutic options not approved by WHO as monotherapy for the treatment of CL (i.e., imiquimod cream, GM-CSF, pentoxifylline, topical honey, etc) [27].

The advantages offered by this combination are that: a) it uses two approaches that are currently recommended for individual use for which there is good information regarding their efficacy and safety when used alone; b) the use of a local and a systemic treatment would hypothetically have an additive effect, since systemic treatment would eliminate circulating or remaining parasites located in the periphery of the lesion, which might be the cause of relapse, that local treatment fails to remove [28]; c) it offers the opportunity to increase the current cure rate reported with any other available treatment approach when used alone; d) the length of treatment with miltefosine is shorter, hopefully reducing the cost and rate of AE associated with 28 days of treatment with miltefosine.

Our results confirmed the superiority of the TT + MLT combination over TT alone for the treatment of uncomplicated CL cases in NW. In the ITT analysis, initial cure was observed in 37 out of 64 (57.8%) and 53 out 66 (80.3%) subjects treated with TT alone or the TT + MLT combination, respectively (p = 0.0055). In the PP analysis, initial cure was achieved by 37 out of 56 (66.1%) and 53 out of 62 (85.5%) subjects in the TT and TT + MLT groups, respectively (p = 0.013). Lesions in subjects treated with the combination healed faster than those in subjects treated with TT alone, 61.2 vs 74.1 days (p = 0.02).

The cure rate observed in the TT alone group was lower than that found in a pooled analysis of eight studies from the Old and New Worlds, which reported an efficacy of 73.2% (95% CI = 69.6–76.7%), however, it is similar to the 64% (95% IC 56–72) cure rate at 3 months reported from Colombia when treating subjects with lesions due to *L*. *panamensis* or *L*. *braziliensis*, as was the case for the subjects included in this study [17,18,29]. In the Old World, similar results have been obtained in Sri Lanka where the cure rate at 3 months in the ITT analysis was 54/98 (55.1%) [30].

A limitation of the present study is the lack of a miltefosine only group in the study design, hence it is not possible to determine with precision how much the addition of TT to a 3-week course of miltefosine increased efficacy. Efficacy results from different studies in the New World using miltefosine at a dose of 2.5mg/kg/day for 28 days vary from 53% to 94% [24,31]. These discrepancies are found even in studies conducted in the same country, since in another study including patients with lesions due to either *L*. *panamensis* or *L*. *braziliensis*, the ITT efficacy of miltefosine was 58.6% (85/145 patients) [4].

To our knowledge only one study was conducted during the early clinical development of miltefosine for CL that used a 3-week regimen. In this study, the per-protocol cure rate of subjects who received a dosage of 50–100 mg/day for 3 weeks was 66%, as compared to 94% in subjects who received 133–150 mg/day for 3–4 weeks [24]. Shorter (one week) or longer (up to 16 weeks) miltefosine regimens have been tested for VL and PKDL respectively, but to our knowledge no other studies have been done in CL patients [32–34].

The disparity in cure rates with miltefosine found in the different clinical trials is probably due to the intrinsic sensitivity to miltefosine of *L*. *braziliensis* strains from different geographical areas. In fact, it has been postulated that some strains of *L*. *braziliensis* may have a reduced capacity to internalize miltefosine from the extracellular medium [35].

For thermotherapy, the most frequently reported AE are consistent with those described in other studies, such as pain at the application site, edema and the presence of vesicles [17–19,36].

In this study, the presence of vesicles at the application site was reported in a significantly higher percentage of subjects compared to previously published studies. This finding can, however, be explained by the safety visit that was carried out 24 hours after the thermotherapy session, which allowed more accurate recording of this AE. Equally, the AEs reported in subjects who received miltefosine were similar to those previously reported (nausea, vomiting and elevation of liver enzymes) and occurred in approximately the same proportion of subjects treated with four weeks of miltefosine [4,20,37–40]. Similarly, no patient discontinued treatment due to gastrointestinal AEs.

Although there have been many advances in the development of new oral treatments for leishmaniasis, most of these efforts have been directed to VL. While patients with CL may also benefit from these efforts, completing the clinical development and registration of any of the potential new treatment candidates under development will require several more years, meaning that currently available treatments for CL probably represent the entire therapeutic arsenal for the coming five to ten years.

We believe there is an opportunity to improve the current treatment recommendations by using a combination of single treatments that are already recommended by WHO and PAHO, but not yet included in many countries’ CL treatment guidelines. This approach could rapidly generate information about the safety and efficacy, and potentially reduce the use of antimonials in patients with uncomplicated CL (subjects with ≤4 lesions of ≤4 cm in diameter and not located on the face or close to mucosal membranes) which represent approximately 60% of all CL cases worldwide. Based on the encouraging results of the present study, we are now planning to conduct a phase III study aiming to determine if this TT + MLT combination is non-inferior to the current recommended first line treatments, meglumine antimoniate (20 mg/kg/day for 20 days parenterally) or miltefosine monotherapy (2.5 mg/kg/day for 28 days orally), for uncomplicated CL cases in the New World.

## Conclusion

the combination of thermotherapy plus a short course of miltefosine showed to be significantly better than thermotherapy alone for the treatment of uncomplicated CL in the New World. In the short-mid- term and until a new, safer and more efficacious treatment become available, the use of combined therapies, using already tested and recommended monotherapies, seems a promising option for patients with uncomplicated CL.

## Supporting information

S1 Checklist Consolidated Standards of Reporting Trials (CONSORT) checklist. (DOC)
